# The occurrence of periodontal diseases and its correlation with different risk factors among a convenient sample of adult Egyptian population: a cross-sectional study

**DOI:** 10.12688/f1000research.20310.2

**Published:** 2020-03-16

**Authors:** Marwa M.S. Abbass, Dina Rady, Israa Ahmed Radwan, Sara El Moshy, Nermeen AbuBakr, Mohamed Ramadan, Nermin Yussif, Ayoub Al Jawaldeh

**Affiliations:** 1 Oral Biology Department, Faculty of Dentistry, Cairo University, Cairo, Egypt; 2 Specialized Dental Hospital, Armed Forces Medical Complex, Kobry El Qobba, Cairo, Egypt; 3 Periodontology Department, MSA University, Cairo, Egypt; 4 University of Vienna, Vienna, A-1090, Austria

**Keywords:** calculus, periodontitis, prevalence, risk factors

## Abstract

**Background**: Even though extensive studies on the prevalence of periodontal diseases in various populations worldwide have been carried out, data for the Egyptian population is limited.  The present study was carried out to evaluate the occurrence and the severity of periodontal disease and its correlation with different risk factors.

**Methods**: Periodontal examination was performed on 343 adults attending the outpatient clinics of the Faculty of Dentistry, Cairo University, as well as three private clinics. Socio-demographic data, brushing frequency, body mass index (BMI) and dietary habits were recorded using a questionnaire.

**Results**: It was found that 58.9% of participants had calculus deposits. The occurrence of periodontitis was 89.8%, where 70.8% of participants had stage I and 15.2% had stage II, while only 4.4% and 2.05% suffered from stage III and stage IV, respectively. Calculus was positively correlated with age, grains, and sugar in drinks and negatively correlated with socioeconomic status, education level, brushing frequency and milk. Calculus was not correlated with gender and BMI. Periodontitis was positively correlated with age, carbohydrates other than bread, grains, and crackers, as well as caffeinated drinks, while negatively correlated with gender, socioeconomic status, brushing frequency. Periodontitis was not correlated with BMI or education level.

**Conclusion**: The present study clarifies that age, brushing frequency, carbohydrates and caffeinated drinks consumption are significant factors influencing the occurrence and the severity of periodontal diseases.

## Introduction

Periodontitis is defined as a chronic, progressive inflammatory disease affecting the periodontium surrounding the tooth. It eventually results in deterioration of the tooth-supporting apparatus and may result in tooth loss if untreated ^1^.

Periodontal diseases, as well as dental caries, are considered the most widespread oral diseases worldwide ^2, 3^. It has been estimated that about 20–50% of the entire global population suffers from periodontal disease ^4^. Residents of developing countries are more prone to periodontal diseases as compared to those of developed countries due to lack of awareness, lack of proper oral hygiene measures, a relatively expensive dental care system and lower socioeconomic status (SES) ^2^.

Periodontal diseases have been linked to increased incidence of multiple systemic diseases such as cardiovascular diseases, metabolic diseases, possible complications of pregnancy, rheumatoid arthritis, respiratory diseases and kidney diseases ^5^. Moreover, periodontal diseases have been also associated with increased risk of malignancies of the oral cavity as well as other sites ^6^.

In 2014, the WHO reported a high prevalence of periodontal diseases in Egypt, 80% of the studied subjects suffered from periodontal diseases ^7^. Despite the high prevalence of periodontal diseases in the Egyptian population, no definite preventive measures are undertaken to screen, prevent or to address this important health issue. Moreover, there is no precedent work correlating the prevalence of periodontal diseases with risk factors including dietary habits in the Egyptian population. Therefore, the aim of the present study is to investigate the incidence of periodontal diseases in correlation with the risk factors amongst a convenient sample from the Egyptian population.

## Methods

### Study design and participants

This study was carried out according to the regulations of the Ethics Committee, Faculty of Dentistry, Cairo University, Egypt (approval: 171217). Convenience sample was utilized in this study. Eligible patients were recruited according to the inclusion and exclusion criteria over a period of two months, starting from the 16th of August 2018 until the 18th of October 2018. Patients were recruited from the outpatient clinics at the Faculty of Dentistry, Cairo University, as well as three private dental offices (Cairo Dental Clinic, Specialized Dental Clinic and El-Rahamn Medical Center). Patients were asked directly to participate in the study while they were attending the clinics. Written consent was obtained from the patients to perform the examinations and for the use and publication of their anonymized data. The inclusion criteria were as follows: age: 18–74 years; gender: males and females; ethnicity: Egyptians. Exclusion criteria were smokers; previous history of/current radiotherapy and/or chemotherapy; pregnant or lactating females; edentulous patients; patients undergoing orthodontic therapy; patients with aggressive periodontitis; patients who had undergone periodontal treatment (including prophylaxis) and/or antibiotic therapy over the past three months.

### Sample size calculation

According to the following simple formula ^8^: $n'\;=\;\frac{NZ^{2}P(1–P)}{d^{2}(N–1)+Z^{3}P(1–P)}$

Where n' = sample size with finite population correction, N = population size, Z = Z statistic for a level of confidence which is conventional (Z value is 1.96), p = expected prevalence and d = precision (5%, d = 0.05). The sample size was estimated to be 339 as the population of Egypt was considered to be 90,000,000, as estimated by the World Bank. The prevalence was estimated to be 32% by averaging the prevalence in India and Bangladesh of 17.5 –21.4% ^9^ and 45% in India ^10^.

### Data collection and grouping

Data were collected using a questionnaire that has previously been used in other studies ^11, 12^ with questions on age, sex, occupation, address, level of education and dietary habits (provided as *Extended data*) ^13^. The questionnaire was filled out by the examiners in the clinics. A Beurer scale (Ulm, Germany) was used to measure body weights with individuals wearing clothing but no shoes. Standing heights were obtained. Body mass index (BMI) was calculated from measured height and weight data. Subjects were classified into the following groups: underweight (BMI<18.5 kg/m ^2^); normal weight (BMI 18.5–24.9 kg/m ^2^); overweight (BMI 25.0–29.9 kg/m ^2^); obese (BMI ≥30.0 kg/ m ^2^). Moreover, patients were categorized into low, moderate and high socioeconomic subgroups based on their education level, occupation, address and the health center where they received their treatments according to a validated socioeconomic status scale for health research in Egypt ^14^.

### Oral examination

Clinical and radiographic case identification was performed by trained examiners (MM and NY) to reach a consensus according to the latest classification of periodontal diseases that was described in 2018 ^15, 16^. The clinical outcomes were the assessment of the presence or absence of calculus and the stage of periodontitis. In order to define the stage of periodontitis, pocket depth (PD) and clinical attachment level (CAL) were measured using a Williams periodontal probe. Periodontitis was categorized into four stages ( Table 1) ^16^.

**Table 1.** Classification of periodontal diseases into four stages ^16^.

Periodontitis stage		Stage I	Stage II	Stage III	Stage IV
Severity	Interdental CAL at site of greatest loss	1–2 mm	3–4 mm	≥5mm	≥5mm
	Radiographic bone loss	Coronal third (<15%)	Coronal third (15% to 33%)	Extending to middle or apical third of the root	
	Tooth loss	No tooth loss due to periodontitis		Tooth loss due to periodontitis of ≤4 teeth	Tooth loss due to periodontitis of ≤5 teeth
Complexity	Local	- Maximum probing depth ≤4mm. - Mostly horizontal bone loss	- Maximum probing depth ≤5mm. - Mostly horizontal bone loss	In addition to Stage II complexity: - Probing depth ≥6mm. - Vertical bone loss ≥3mm. - Furcation involvement class II or III - Moderate ridge defect	In addition to Stage III complexity: Need for complex rehabilitation due to: - Masticatory dysfunction - Secondary occlusal trauma (tooth mobility degree ≥2) - Severe ridge defect - Bite collapse, drifting, flaring - Less than 20 remaining teeth (10 opposing pairs)

CAL, clinical attachment loss.

### Statistical analysis

Data were statistically described in terms of number of cases and percentages. Comparison between the study groups was done using ANOVA test with post-hoc multiple two-group comparisons. For comparing categorical data, Chi-square (χ ^2^) test was performed. Correlation between variables was done using Spearman rank correlation equation. *p* values <0.05 were considered statistically significant. All statistical calculations were done using IBM SPSS (Statistical Package for the Social Science; IBM Corp, Armonk, NY, USA) release 22 for Microsoft Windows.

## Results

### Population profile

**Figure 1.**
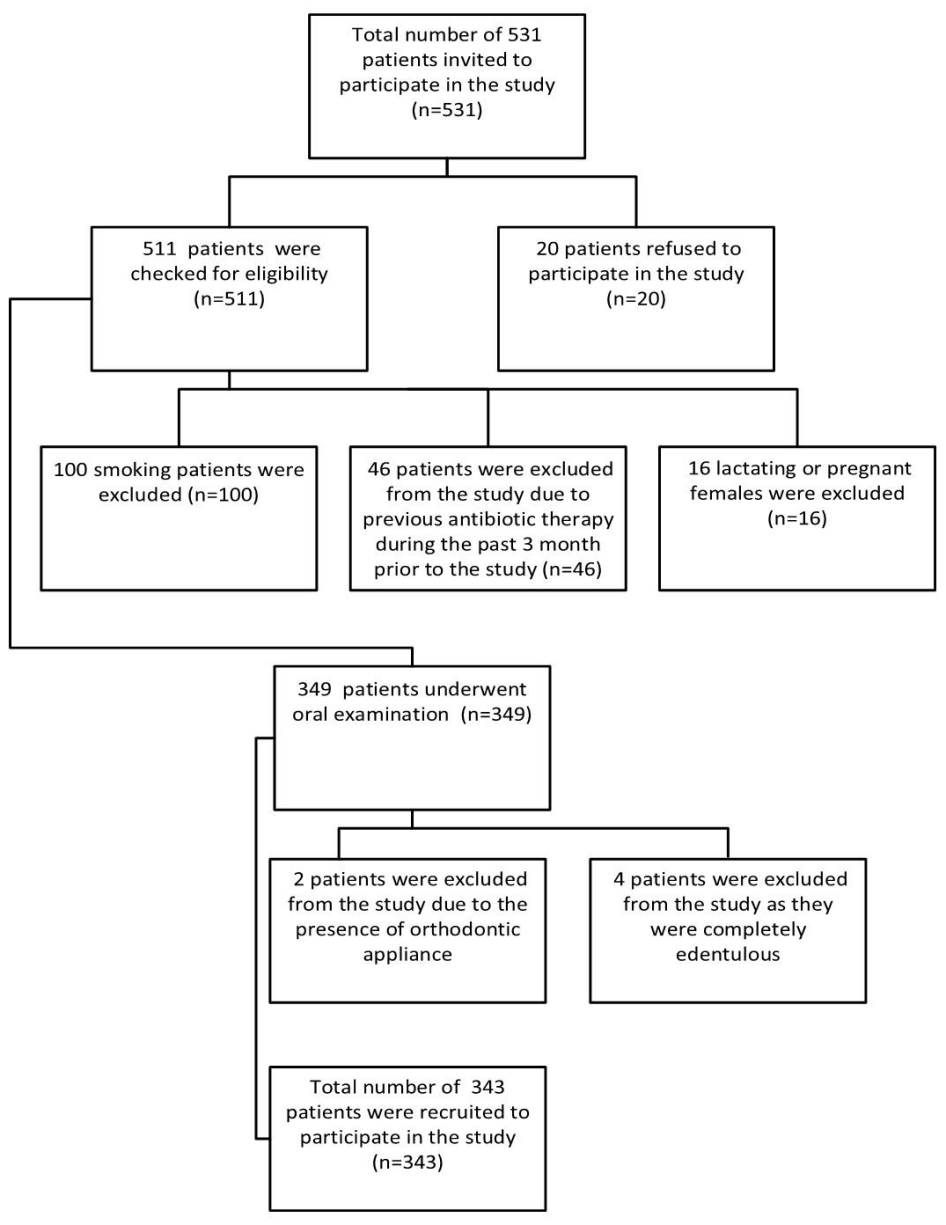
Flow chart of patient selection.

The number of individuals at each stage of the study are shown in Figure 1 and the number and percentage of patients in different categories are presented in Table 2. It was found that 24.5% of participants brush their teeth twice daily, while 23.3% don’t brush their teeth ^17^. The occurrence of calculus was 58.9%. The occurrence of periodontitis was 89.8%, where 70.8.5% of participants had stage I periodontitis and 15.2% had stage II, while 4.4.% and 2.04% of participants had stage III and stage IV, respectively.

**Table 2.** Descriptive analysis of categorical variables (N=343).

Parameter	Categories, number (%)					
**1. Age**	**18–34 years**		**35–49 years**		**50–70 years**	
	176 (51.3)		104 (30.3)		60 (17.5)	
**2. Gender**	**Males**			**Females**		
	139 (40.5)			204 (59.5)		
**3. Body Mass Index**	**Underweight**	**Normal**		**Overweight**	**Obese**	
	2 (0.6)	127 (37.0)		135 (39.4)	79 (23.0)	
**4. Socioeconomic status**	**Low**		**Moderate**		**High**	
	94 (27.4)		142 (41.4)		107 (31.2)	
**5. Level of education**	**Low**		**Moderate**		**High**	
	50 (14.6)		116 (33.8)		177 (51.6)	
**6. Biological risk factors**						
** Brushing frequency**	**No brushing**	**Infrequent**	**Once daily**	**Twice daily**	**Three times a day**	
	80 (23.3)	45 (13.1)	113 (32.9)	84 (24.5)	21 (6.1)	
** Reasons for not** **brushing**	**Bleeding**	**I don't know** **how to brush**	**I forget**	**I don't have** **time**	**Other**	
	23 (6.7)	7 (2.0)	23 (6.7)	16 (4.7)	11(3.2)	
**7. Dietary habits**	**≤ 2 times/week**		**3–6 times/week**		**1–6 times/day**	
** Bread**	16 (4.7)		16 (4.7)		311 (90.7)	
** Other carbohydrates**	74 (21.6)		43 (12.5)		226 (65.9)	
** Eggs**	194 (56.6)		56 (16.3)		92 (26.8)	
** Fruits/vegetables**	68 (19.8)		56 (16.3)		219 (63.8)	
** Milk**	183 (53.4)		22 (6.4)		138 (40.2)	
** Milk products**	97 (28.3)		44 (12.8)		202 (58.9)	
** Grains**	133 (38.8)		51 (14.9)		156 (45.5)	
** Sugars in beverages**	62 (18.1)		14 (4.1)		266 (77.6)	
** Sugars not in** **beverages**	229 (66.8)		22 (6.4)		91 (26.5)	
** Jam, molasses and** **honey**	248 (72.3)		34 (9.9)		61 (17.8)	
** Candies**	233 (67.9)		38 (11.1)		71 (20.7)	
** Crackers**	176 (51.3)		35 (10.2)		131 (38.2)	
** Junk food**	207 (60.3)		34 (9.9)		101 (29.4)	
** Chocolate**	250 (72.9)		33 (9.6)		60 (17.5)	
** Soda**	198 (57.7)		43 (12.5)		102 (29.7)	
** Juices**	209 (60.9)		33 (9.6)		101 (29.4)	
** Citrus juices**	263 (76.7)		26 (7.6)		54 (15.7)	
** Caffeinated drinks**	41 (12.0)		16 (4.7)		286 (83.4)	
**7. Calculus**	**Yes**			**No**		
	202 (58.9)			141 (41.1)		
**8. Periodontitis**	**No** **periodontitis**	**Stage I**	**Stage II**	**Stage III**	**Stage IV**	**Total** **periodontitis**
	35 (10.2)	234 (70.8)	52 (15.2)	15 (4.4)	7 (2.04)	308 (89.8)

### Correlation between calculus and different risk factors

As shown in Table 3, the highest percentage of calculus among different age groups was recorded in adults aged 50–70 years (70%). A comparison of the occurrence of calculus between age subgroups revealed a statistically significant difference (p =0.001). There was a positive correlation between age and calculus (rho=-0.192, p <0.001).

**Table 3.** Correlation of calculus and periodontitis with different risk factors (N=343).

Parameters and categories		Number (%)		Correlation		Pearson’s Chi- square	Number (%)					Correlation		Pearson’s Chi- square
		Calculus		rho	p-value	p-value	Periodontitis					rho	p-value	p-value
		Yes	No				None	Stage I	Stage II	Stage III	Stage IV			
**1. Age**	**18–34 years**	87 (49.4)	89 (50.6)	0.192	<0.001 *	0.001 *	25 (14.2)	124 (70.5)	20 (11.4)	5 (2.8)	2 (1.1)	0.206	<0.001	0.005 *
	**35–49 years**	71 (68.3)	33 (31.7)				8 (7.7)	69 (66.3)	18 (17.3)	4 (3.8)	5 (4.8)			
	**50–70 years**	42 (70.0)	18 (30.0)				2 (3.3)	38 (63.3)	14 (23.3)	6 (10.0)	0 (0.0)			
**2. Gender**	**Males**	89 (64.0)	50 (36.0)	-0.086	0.111	0.110	11 (7.9)	90 (64.7)	24 (17.3)	9 (6.5)	5 (3.6)	-0.129	0.017 *	0.115
	**Females**	113 (55.4)	91 (44.6)				24 (11.8)	144 (70.6)	28 (13.7)	6 (2.9)	2 (1.0)			
**3. Body Mass** **Index**	**Underweight**	2 (100.0)	0 (0.0)	0.101	0.062	0.129	0 (0.0)	2 (100.0)	0 (0.0)	0 (0.0)	0 (0.0)	0.081	0.137	0.494
	**Normal**	66 (52.0)	61 (48.0)				16 (12.6)	88 (69.3)	20 (15.7)	2 (1.6)	1 (0.8)			
	**Overweight**	82 (60.7)	53 (39.3)				14 (10.4)	86 (63.7)	24 (17.8)	8 (5.9)	3 (2.2)			
	**Obese**	52 (65.8)	27 (34.2)				5 (6.3)	58 (73.4)	8 (10.1)	5 (6.3)	3 (3.8)			
**4.** **Socioeconomic** **status**	**Low**	68 (72.3)	26 (27.7)	-0.254	<0.001 *	<0.001 *	5 (5.3)	64 (68.1)	16 (17)	6 (6.4)	3 (3.2)	-0.176	0.001 *	0.072
	**Moderate**	91 (64.1)	51 (35.9)				13 (9.2)	94 (66.2)	23 (16.2)	8 (5.6)	4 (2.8)			
	**High**	34 (40.2)	64 (59.8)				17 (15.9)	76 (71.0)	13 (12.1)	1 (0.9)	0 (0.0)			
**5. Level of** **education**	**Low**	40 (80.0)	10 (20.0)	-0.167	0.002 *	0.002 *	1 (2.0)	31 (62.0)	9 (18.0)	7 (14.0)	2 (4.0)	-0.009	0.067	0.001 *
	**Moderate**	69 (59.5)	47 (40.5)				9 (7.8)	91 (78.4)	14 (12.1)	1 (0.9)	1 (0.9)			
	**High**	93 (52.5)	84 (47.5)				25 (14.1)	112 (63.3)	29 (16.4)	7 (4.0)	4 (2.3)			
**6. Biological risk factors**														
**Brushing** **frequency**	**No brushing**	63 (78.8)	17 (21.2)	-0.326	<0.001 *	<0.001 *	0 (0.0)	53 (66.3)	15 (18.8)	9 (11.3)	3 (3.8)	-0.234	<0.001 *	0.003 *
	**Infrequent**	34 (75.6)	11 (24.4)				3 (6.7)	29 (64.4)	8 (17.8)	2 (4.4)	3 (6.7)			
	**Once daily**	35 (57.5)	48 (42.5)				17 (15.0)	79 (69.9)	13 (11.5)	3 (2.7)	1 (0.9)			
	**Twice daily**	33 (39.3)	51 (60.7)				12 (14.3)	58 (69.0)	13 (15.5)	1 (1.2)	0 (0.0)			
	**Three times**	7 (33.3)	14 (66.7)				3 (14.3)	15 (71.4)	3 (14.3)	0 (0.0)	0 (0.0)			
**7. Dietary habits**														
**Bread**	**≤ 2 times/week**	17 (87.5)	2 (12.5)	-0.031	0.566	0.015 *	1 (6.3)	12 (75.0)	1 (6.3)	0 (0.0)	2 (12.5)	0.041	0.448	0.104
	**3–6 times/week**	6 (37.5)	10 (62.5)				3 (18.8)	11 (68.8)	2 (12.5)	0 (0.0)	0 (0.0)			
	**1–6 times/day**	182 (58.5)	129 (41.5)				31 (10.0)	211 (67.8)	49 (15.8)	15 (4.8)	5 (1.6)			
**Other** **carbohydrates**	**≤ 2 times/week**	45 (60.8)	29 (39.2)	0.022	0.685	0.357	8 (10.8)	57 (77.0)	6 (8.1)	1 (1.4)	2 (2.7)	0.142	0.008 *	0.046 *
	**3–6 times/week**	21 (48.8)	22 (51.2)				9 (20.9)	26 (60.5)	7 (16.3)	0 (0.0)	1 (2.3)			
	**1–6 times/day**	136 (60.2)	39 (39.8)				18 (8.0)	151 (66.8)	39 (17.3)	14 (6.2)	4 (1.8)			
**Eggs**	**≤ 2 times/week**	119 (61.3)	75 (38.7)	-0.072	0.182	0.322	18 (9.3)	140 (72.2)	28 (14.4)	7 (3.6)	1 (0.5)	0.006	0.908	0.045 *
	**3–6 times/week**	34 (60.7)	22 (39.3)				4 (7.1)	31 (55.4)	15 (26.8)	4 (7.1)	2 (3.6)			
	**1–6 times/day**	48 (52.2)	44 (47.8)				13 (14.1)	63 (68.5)	9 (9.8)	4 (4.3)	3 (3.3)			
**Fruits/** **vegetables**	**≤ 2 times/week**	47 (69.1)	21 (30.9)	-0.069	0.205	0.791	5 (7.4)	49 (72.1)	11 (16.2)	1 (1.5)	2 (2.9)	0.058	0.283	0.410
	**3–6 times/week**	30 (53.6)	26 (46.4)				10 (17.9)	38 (67.9)	5 (8.9)	2 (3.6)	1 (1.8)			
	**1–6 times/day**	125 (57.1)	94 (42.9)				20 (9.1)	147 (67.1)	36 (16.4)	12 (5.5)	4 (1.8)			
**Milk**	**≤ 2 times/week**	119 (65.0)	64 (33.0)	-0.133	0.013 *	0.046 *	18 (9.8)	119 (65.0)	32 (17.5)	9 (4.9)	5 (2.7)	-0.076	0.16	0.308
	**3–6 times/week**	12 (54.5)	10 (45.5)				1 (4.5)	19 (86.4)	0 (0.0)	2 (9.1)	0 (0.0)			
	**1–6 times/day**	71 (51.4)	67 (48.6)				16 (11.6)	96 (69.6)	20 (14.5)	4 (2.9)	2 (1.4)			
**Milk products**	**≤ 2 times/week**	59 (60.8)	38 (39.2)	-0.017	0.753	0.884	9 (9.3)	65 (67.0)	12 (12.4)	8 (8.2)	3 (3.1)	-0.048	0.38	0.353
	**3–6 times/week**	25 (56.8)	19 (43.2)				4 (9.1)	31 (70.5)	6 (13.6)	1 (2.3)	2 (4.5)			
	**1–6 times/day**	118 (58.4)	84 (41.6)				22 (10.9)	138 (68.3)	34 (16.8)	6 (3.0)	2 (1.0)			
**Grains**	**≤ 2 times/week**	68 (5.1)	65 (48.9)	0.133	0.014 *	0.049 *	18 (13.5)	99 (74.4)	12 (9.0)	4 (3.0)	0 (0.0)	0.181	0.001 *	0.021 *
	**3–6 times/week**	30 (58.8)	21 (41.2)				6 (11.8)	31 (60.8)	8 (15.7)	3 (5.9)	3 (5.9)			
	**1–6 times/day**	102 (65.4)	54 (34.6)				11 (7.1)	102 (65.4)	32 (20.5)	7 (4.5)	4 (2.6)			
**Sugar in drinks**	**≤ 2 times/week**	29 (46.8)	33 (53.2)	0.139	0.010 *	0.032 *	11 (17.7)	42 (67.7)	5 (8.1)	2 (3.2)	2 (3.2)	0.105	0.053	0.170
	**3–6 times/week**	6 (42.9)	8 (57.1)				2 (14.3)	8 (57.1)	2 (14.3)	2 (14.3)	0 (0.0)			
	**1–6 times/day**	167 (62.8)	99 (37.2)				22 (8.3)	183 (68.8)	45 (16.9)	11 (4.1)	5 (1.9)			
**Sugar not in** **drinks**	**≤ 2 times/week**	143 (62.4)	68 (37.6)	-0.081	0.132	0.031 *	23 (10.0)	147 (64.2)	41 (17.9)	12 (5.2)	6 (2.6)	-0.114	0.034 *	0.579
	**3–6 times/week**	7 (31.8)	15 (68.2)				4 (18.2)	14 (63.6)	3 (13.6)	1 (4.5)	0 (0.0)			
	**1–6 times/day**	51 (56.0)	40 (44.0)				8 (8.8)	72 (79.1)	8 (8.8)	2 (2.2)	1 (1.1)			
**Jam, molasses** **and honey**	**≤ 2 times/week**	146 (58.9)	102 (41.1)	0.001	0.988	1.000	23 (9.3)	171 (69.0)	39 (15.7)	10 (4.0)	5 (2.0)	-0.026	0.626	0.774
	**3–6 times/week**	20 (58.8)	14 (41.2)				6 (17.6)	20 (58.8)	4 (11.8)	3 (8.8)	1 (2.9)			
	**1–6 times/day**	36 (59.0)	25 (41.0)				6 (9.8)	43 (70.5)	9 (14.8)	2 (3.3)	1 (1.6)			
**Candies**	**≤ 2 times/week**	144 (61.8)	89 (38.2)	-0.067	0.215	0.135	29 (12.4)	157 (67.4)	35 (15.0)	8 (3.4)	4 (1.7)	0.105	0.052	0.166
	**3–6 times/week**	17 (44.7)	21 (55.3)				4 (10.5)	25 (65.8)	5 (13.2)	4 (10.5)	0 (0.0)			
	**1–6 times/day**	41 (57.7)	30 (42.3)				2 (2.8)	51 (71.8)	12 (16.9)	3 (4.2)	3 (4.2)			
**Crackers**	**≤ 2 times/week**	104 (59.1)	72 (40.9)	0.020	0.714	0.366	26 (14.8)	115 (65.3)	27 (15.3)	5 (2.8)	3 (1.7)	0.111	0.040 *	0.168
	**3–6 times/week**	17 (48.6)	18 (51.4)				1 (2.9)	26 (74.3)	6 (17.1)	2 (5.7)	0 (0.0)			
	**1–6 times/day**	81 (61.8)	50 (38.2)				8 (6.1)	92 (70.2)	19 (14.5)	8 (6.1)	4 (3.1)			
**Junk food**	**≤ 2 times/week**	122 (58.9)	85 (41.1)	0.009	0.875	0.897	25 (12.1)	136 (65.7)	32 (15.5)	11 (5.3)	3(1.4)	0.01	0.853	0.641
	**3–6 times/week**	19 (55.9)	15 (44.1)				1 (2.9)	24 (70.6)	7 (20.6)	1 (2.9)	1 (2.9)			
	**1–6 times/day**	61 (60.4)	40 (39.6)				9 (8.9)	73 (72.3)	13 (12.9)	3 (3.0)	3 (3.0)			
**Chocolate**	**≤ 2 times/week**	146 (58.4)	104 (41.6)	0.023	0.673	0.680	28 (11.2)	170 (68.0)	36 (14.4)	11 (4.4)	5 (2.0)	0.036	0.507	0.579
	**3–6 times/week**	18 (54.5)	15 (45.5)				2 (6.1)	20 (60.6)	7 (21.2)	2 (6.1)	2 (6.1)			
	**1–6 times/day**	38 (63.3)	22 (36.6)				5 (8.3)	44 (73.3)	9 (15.0)	2 (3.3)	0 (0.0)			
**Soda**	**≤ 2 times/week**	121 (61.1)	77 (38.9)	-0.066	0.225	0.339	23 (11.6)	135 (68.2)	30 (15.2)	9 (4.5)	1 (0.5)	0.036	0.51	<0.001 *
	**3–6 times/week**	27 (62.8)	16 (37.2)				3 (7.0)	26 (60.5)	4 (9.3)	4 (9.3)	6 (14.0)			
	**1–6 times/day**	54 (52.9)	48 (47.1)				9 (8.8)	73 (71.6)	18 (17.6)	2 (2.0)	0 (0.0)			
**Juices**	**≤ 2 times/week**	123 (58.9)	86 (41.4)	0.008	0.886	0.839	25 (12.0)	142 (67.9)	25 (12.0)	12 (5.7)	5 (2.4)	0.074	0.17	0.112
	**3–6 times/week**	18 (54.5)	15 (45.5)				4 (12.1)	24 (72.7)	3 (9.1)	1 (3.0)	1 (3.0)			
	**1–6 times/day**	61 (60.4)	40 (39.6)				6 (5.9)	68 (67.3)	24 (23.8)	2 (2.0)	1 (1.0)			
**Citrus juices**	**≤ 2 times/week**	160 (60.8)	103 (39.2)	-0.075	0.163	0.335	27 (10.3)	178 (67.7)	40 (15.2)	14 (5.3)	4 (1.5)	-0.023	0.666	0.046 *
	**3–6 times/week**	15 (57.7)	11 (42.3)				2 (7.7)	17 (65.4)	4 (15.4)	0 (0.0)	3 (11.5)			
	**1–6 times/day**	27 (50.0)	27 (50.0)				6 (11.1)	39 (72.2)	8 (14.8)	1 (1.9)	0 (0.0)			
**Caffeinated** **drinks**	**≤ 2 times/week**	19 (46.3)	22 (53.7)	0.091	0.091	0.206	4 (9.8)	33 (80.5)	4 (9.8)	0 (0.0)	0 (0.0)	0.114	0.034 *	0.189
	**3–6 times/week**	9 (56.3)	7 (43.7)				1 (6.3)	15 (93.8)	0 (0.0)	0 (0.0)	0 (0.0)			
	**1–6 times/day**	174 (60.8)	112 (39.2)				30 (10.5)	186 (65.0)	48 (16.8)	15 (5.2)	7 (2.4)			

The correlation coefficient, rho, ranges from -1 to +1, where 1 = perfect positive correlation, 0 = no correlation, -1 = perfect negative (inverse) correlation. *Statistical significance at p-value < 0.05.

Regarding gender and BMI, males and obese adults had the highest incidence of calculus (55.4% and 65.8%, respectively). A comparison of the occurrence of calculus between gender subgroups as well as a comparison between BMI subgroups were statistically insignificant (p ≥0.05). There was no correlation between either of these factors and calculus (rho=-0.086, p=0.111 and rho=-0.101, p =0.062, respectively). 

Regarding SES, education level and brushing frequency, adults with a low SES, a low educational level and those who don’t brush their teeth had the highest occurrence of calculus (72.3%, 80% and 78.8%, respectively). A comparison of calculus occurrence between SES, education level and brushing frequency subgroups revealed a statistically significant difference (p=<0.05) and there was an inverse correlation between these factors and calculus (rho=-0.254, p<0.001; rho=-0.167, p=0.002; and rho=-0.326, p <0.001, respectively). 

Adults who consume bread, carbohydrates other than bread, eggs, fruits and vegetables, milk, milk products, candies and citrus juices less than or equal to two times a week had the highest occurrence of calculus compared to those who consumed these products more frequently (87.5%, 60.8%, 61.3%, 69.1%, 65%, 60.8%, 61.8% and 60.8%, respectively). Those who consume grains, sugars in drinks, sugar not in drinks, jams, crackers, junk food, chocolates, juices and caffeinated drinks with a frequency of one to six times per day had the highest occurrence of calculus (65.4%, 62.8%, 65.1%, 59%, 61.8%, 60.4%, 63.3%, 60.4% and 60.8%, respectively), as well as those who consume soda three to six times per week (62.8%).

A comparison of calculus occurrence between consumption frequency subgroups for all dietary elements was statistically insignificant except for milk, grains and sugars in drinks (p<0.05). There was a positive correlation between consumption frequency of grain, sugars in drinks and calculus (rho=0.133, p=0.014 and rho=0.139, p=0.010, respectively), while milk revealed an inverse correlation (rho= -0.133, p =0.013).

### Correlation of periodontitis and different risk factors

As it is revealed in Table 3, the highest occurrence of periodontitis among different age groups (96.7%) was recorded among adults aged (50–70 years). In all age groups, the majority of participants suffered from stage I periodontitis: 70.5% of adults aged 18–34 years; 66.3% of adults aged 35–49 years; and 63.3% of adults aged 50–70 years. A comparison of periodontitis occurrence between age subgroups revealed a statistically significant difference (p=0.005). There was a positive correlation between age and periodontitis (rho=0.206, p=<0.001).

The highest percentage of periodontitis was recorded among males (92.1%), while in females the occurrence was 88.2 %. Stage I periodontitis was predominant, with 64.7% of males and 70.6% of females with this stage of periodontitis. A comparison of periodontitis occurrence between gender subgroups showed a statistically insignificant difference (p=0.115). There was a correlation between male gender and periodontitis (rho= -0.129, p=0.017). 

Among different BMI groups, the highest occurrence of periodontitis was among obese participants (93.7%). Stage I periodontitis was the predominate stage, with 100% of underweight, 69.3% of normal, 63.7% of overweight adults and 73.4% of obese participants in this stage of the disease. A comparison of periodontitis occurrence between BMI subgroups revealed a statistically insignificant difference (p≥0.05). There was no correlation between BMI and periodontitis (rho=0.081, p=0.137). 

Regarding SES and education levels, participants with a low SES and a low educational level had the highest occurrence of periodontitis (94.7% and 98%, respectively). In all SES and education level subgroups, most participants had stage I periodontitis ( Table 3). A comparison of periodontitis between SES subgroups revealed a statistically insignificant difference (p ≥0.05) while there was a statistically significant difference (p =0.001) between education level subgroups. There was no correlation between periodontitis and education level (rho= -0.009, p=0.067), while an inverse correlation was found between periodontitis and SES (rho=-0.176, p =0.001). 

In the present study, all adults who reported that they don’t brush their teeth had periodontitis (100%). The majority of participants in all brushing frequency subgroups suffered from stage I periodontitis ( Table 3). A comparison of periodontitis occurrence between subgroups revealed a statistically significant difference (p=0.003). There was an inverse correlation between brushing frequency and periodontitis (rho= -0.234, p = <0.001). 

A comparison of periodontitis incidence between consumption frequency subgroups for all dietary elements was statistically insignificant except for the consumption of other carbohydrates, eggs, grains, soda and citrus juices (p>0.05). The consumption frequencies of carbohydrates other than bread, grains, crackers and caffeinated drinks were shown to have a positive correlation with periodontitis (rho=0.142, p=0.008; rho=0.181, p=0.001; rho=0.111, p=0.04; and rho=0.114, p=0.034, respectively). Moreover, the consumption frequencies of sugar in drinks and candies were very close to a significant positive correlation with periodontitis (rho=0.105, p=0.053 and rho=0.105, p=0.052, respectively). For the consumption of all foods at all frequencies, the majority of participants suffered from stage I periodontitis ( Table 3).

## Discussion

Surveying the prevalence of periodontal diseases is challenging because of case misclassification and the number of teeth and sites to be examined ^18^. According to the Canadian Health Measures Survey, the measurement of periodontal ligament attachment loss is the gold standard in reporting the prevalence of periodontal disease ^19^.

In the current study, a new classification was utilized, where periodontitis is graded into stages according to the severity as well as the complexity of the treatment required to eliminate local risk factors. This classification is advantageous over others as it gives an idea about the severity, diagnosis, pathogenesis and the required treatment of periodontal conditions ^15, 16^.

In this study, positive correlations were found between calculus, periodontitis, and age. It is well established that periodontal destruction is associated with periodontal disease activity, which is cumulative and tends to increase with age ^20^.

Male gender was correlated with the severity of periodontitis in the present investigation. Similar findings have been reported in a previous study conducted in southern Thailand ^21^. This could be attributed to neglected oral-hygiene measures in males. Moreover, sex differences in periodontal disease may be due to gender-based heterogeneity in immune responses ^22^.

A negative correlation was found in the current study between periodontal health and SES, as well as a negative correlation was detected between the level of education and calculus among the studied participants. Other authors concur SES ^23, 24^, and education ^23, 25^, among other factors, that are influential on oral and periodontal health. Patients with low SES usually lack proper dental education, fail to visit the dentist on a regular basis and usually seek the dentist only in case of symptomatic complains ^24^. The level of individual education is a component of SES. Individuals with higher education levels usually have a higher income and higher SES and are more likely to have routine, prophylactic dentist visits ^26^. Moreover, education level influences the patient’s oral hygiene practice and dietary habits ^27^. These factors and their associated psychological stresses negatively impact oral health through increasing inflammatory mediators and stimulating inflammation and altering host immune response to bacterial insult ^28^.

Another risk factor for periodontal disease is poor oral hygiene, associated with the accumulation of plaque and calculus that result in gingivitis, which eventually results in periodontitis if untreated ^29^. This is in accordance with the findings of the current study, which revealed a negative correlation between the frequency of teeth brushing and the presence of calculus and periodontitis.

Although the influence of dietary habits on dental caries is more significant as compared to their influence on periodontal disease; nonetheless, a poor diet can negatively affect periodontal tissues, causing rapid progression of periodontal disease ^30^. Malnutrition can modulate the inflammatory process and immune response ^28^, which subsequently may cause periodontal disease ^31^. One proposed mechanism through which nutrition can influence periodontal health is reactive oxygen species (ROS) and oxidative stresses. The presence of excessive oxidants can result in tissue damage via oxidation of important molecules, production of pro-inflammatory mediators as well as local and systematic inflammation, which negatively affects periodontal health ^31^.

Many dietary components, such as fats and sugars, can cause oxidative stress and increased ROS production, which promotes inflammatory processes ^32^ and negatively impacts periodontal health ^33^. Additionally, a sugary diet is linked to increased plaque formation. This could explain the positive correlation observed in the current study between sugar in drinks and calculus deposits and between intake of carbohydrates other than bread and crackers and periodontitis. Similar linkage between a high sugary diet and increased risk of periodontal disease and calculus deposits have been reported in previous studies ^33^.

In the present work, a negative correlation between calculus and milk consumption has been reported. These results support the findings of Adegboye *et al.*, who reported that dairy calcium, particularly from milk, is associated with a reduced risk of periodontitis ^34^.

Heavy coffee consumption was linked to an increased risk of periodontitis in the Korean population ^35^. Likewise, a positive correlation was detected between the consumption of caffeinated drinks and periodontitis in the current study. This can be ascribed to their sugar as well as their caffeine content. Caffeine has been reported to increase alveolar bone loss in rats with induced periodontitis and reduce bone healing following teeth extraction ^36^. Caffeine can enhance osteoclastic activity and suppress osteoblasts proliferation ^37^. On the contrary, Machida *et al.* ^38^ reported an inverse association between coffee consumption and periodontitis. The discrepancy in the reported effect of coffee on the alveolar bone can be attributed to different dosages of coffee and caffeine administered in each experiment.

A healthy diet rich in fibers and whole grains intake is associated with reduced risk of periodontitis in several populations ^39, 40^. This is owing to the health benefits of whole grain, as they are rich in antioxidants and fibres ^41^. Antioxidant intake has been positively associated with periodontal health ^30, 42^.

In the current study, a positive association between grain intake and periodontitis was observed. According to Hassan-Wassef ^43^, the most commonly consumed grain in Egypt is fava beans. In the Egyptian cuisine, dried fava beans are slowly stewed overnight before being served. Therefore, it could be deduced that the boiling of beans has a negative impact on its antioxidant content ^44^. Moreover, they are usually served alongside bread and combined with unsaturated fats and oils and many Egyptians consume fava beans from street vendors, which could be above-mentioned factors may alter host inflammatory response and negatively impact oral and periodontal health.

Even though the current work investigated the occurrence of periodontal diseases in correlation to different risk factors, important risk factors still need to be investigated such as smoking and glycosylated haemoglobin level.

An important limitation of the current work is the exclusion of patients with aggressive periodontitis where this group of patients together with the severe chronic periodontitis patients represents the individuals in stages III &VI. The low recorded percentages of periodontitis in these two stages could be referred to this exclusion. This consideration should be taken in future studies implementing the new 2018 periodontal classification.

Moreover, among the limitations of the present study is the small and convenient sample recruited from adults attending the free dental clinic at Faculty of Dentistry and three private clinics at great Cairo. Although, a large number of great Cairo residences are internal immigrants from different regions of Egypt, including other geographical regions from Egypt may provide a better and accurate representation of the Egyptian population.

In conclusion, periodontitis is a multifactorial disease with many risk factors. Its progression is dependent on the interaction between intricate parameters, which pave the way to bacteria-induced inflammation and tissue destruction. A proper oral hygiene regime and nutrient-rich healthy diet in addition to prophylactic dental visits can reduce the risk of periodontal diseases and promote oral health.

## Data availability

### Underlying data

Figshare: Raw data for periodontitis 2.xlsx. https://doi.org/10.6084/m9.figshare.9756428.v1 ^17^

### Extended data

Figshare: questionnaire periodontitis adult.docx. https://doi.org/10.6084/m9.figshare.9756542.v1 ^13^

Data are available under the terms of the Creative Commons Zero “No rights reserved“ data waiver (CC0 1.0 Public domain dedication).
