# Supplementary material for: The occurrence of periodontal diseases and its correlation with different risk factors among a convenient sample of adult Egyptian population: a cross-sectional study
Source: F1000Res. 2020 Mar 16;8:1740. Originally published 2019 Oct 11. [Version 2] doi: 10.12688/f1000research.20310.2 (PMC7236581; doi:10.12688/f1000research.20310.2)
Supplement: Peer review history

# Peer review: Reviewer response for version 2

**DOI:** 10.5256/f1000research.25211.r61366

**Published:** 2020-05-18

**Authors:** Reema Fayez Tayyem

**Affiliations:** 1 Department of Nutrition and Food Technology, Faculty of Agriculture, University of Jordan, Amman, Jordan

No more comments.

Is the work clearly and accurately presented and does it cite the current literature?

Yes

If applicable, is the statistical analysis and its interpretation appropriate?

I cannot comment. A qualified statistician is required.

Are all the source data underlying the results available to ensure full reproducibility?

No source data required

Is the study design appropriate and is the work technically sound?

Partly

Are the conclusions drawn adequately supported by the results?

Yes

Are sufficient details of methods and analysis provided to allow replication by others?

Yes

Reviewer Expertise:

NA

I confirm that I have read this submission and believe that I have an appropriate level of expertise to confirm that it is of an acceptable scientific standard.

# Peer review: Reviewer response for version 2

**DOI:** 10.5256/f1000research.25211.r61365

**Published:** 2020-04-22

**Authors:** Enas Elgendy

**Affiliations:** 1 Faculty of Dentistry, Kafr El-Sheikh University, Kafr El Sheikh, Egypt

I read the revised article and, in my opinion, these changes have improved the article. Thus, I accept the revised article.

Is the work clearly and accurately presented and does it cite the current literature?

Yes

If applicable, is the statistical analysis and its interpretation appropriate?

Yes

Are all the source data underlying the results available to ensure full reproducibility?

Yes

Is the study design appropriate and is the work technically sound?

Partly

Are the conclusions drawn adequately supported by the results?

Yes

Are sufficient details of methods and analysis provided to allow replication by others?

Yes

Reviewer Expertise:

Periodontology & Oral medicine

I confirm that I have read this submission and believe that I have an appropriate level of expertise to confirm that it is of an acceptable scientific standard.

# Peer review: Reviewer response for version 1

**DOI:** 10.5256/f1000research.22315.r56359

**Published:** 2019-11-26

**Authors:** Enas Elgendy

**Affiliations:** 1 Faculty of Dentistry, Kafr El-Sheikh University, Kafr El Sheikh, Egypt

This is an interesting study in the field of periodontology as the authors have tried to find the correlation between the common risk factors of periodontitis and the different stages of the diseases among Egyptian patients according to the new classification published in 2018.  

 However, the authors have excluded patients with aggressive periodontitis without justification for their exclusion and this group of patients represents most of patients in stages 3 & 4.

 The results of  the study were low in stage III & VI (4.4% & 2.05 % respectively) and this may be attributed to excluding aggressive periodontitis from the study design. The aggressive periodontitis should be included in the study design because this type with sever chronic periodontitis represent stage III &VI. 

 In addition, the study design did not include diabetes mellitus and smoking among the studied risk factors. One of the strength points of the study is that the national common food and drinks as beans and tea were included among the risk factors.

 This study has not been conducted before in Egyptian patients according to stages and the finding of different risk factors among the Egyptian patients can reduce the prevalence of these diseases in the future.

Is the work clearly and accurately presented and does it cite the current literature?

Yes

If applicable, is the statistical analysis and its interpretation appropriate?

Yes

Are all the source data underlying the results available to ensure full reproducibility?

Yes

Is the study design appropriate and is the work technically sound?

Partly

Are the conclusions drawn adequately supported by the results?

Yes

Are sufficient details of methods and analysis provided to allow replication by others?

Yes

Reviewer Expertise:

Periodontology & Oral medicine

I confirm that I have read this submission and believe that I have an appropriate level of expertise to confirm that it is of an acceptable scientific standard.

# Response

**Published:** 2020-03-11

**Authors:** Marwa Abbass

**Affiliations:** Faculty of Dentistry, Cairo University, Egypt

**Dear reviewer:**

**Thanks for your comprehensive revision of this article, we do really appreciate your comments.**

**These are our responses for your valuable comments**

**Comment 1:**

“However, the authors have excluded patients with aggressive periodontitis without justification for their exclusion and this group of patients represents most of the patients in stages 3 & 4.

The results of the study were low in stage III & VI (4.4% & 2.05 % respectively) and this may be attributed to excluding aggressive periodontitis from the study design. The aggressive periodontitis should be included in the study design because this type with severe chronic periodontitis represents stage III &VI. 

**Answer:**

The authors do appreciate the reviewer’s comment. This is the first study to be conducted using the new periodontal classification, therefore this point despite its importance has not been taken into consideration as usually in previous studies following the old classification the aggressive periodontitis patient was excluded.  A limitation paragraph has been added in the discussion section to clarify this valuable point for further research implementation.

**Revised Text:**

An important limitation of the current work is the exclusion of patients with aggressive periodontitis where this group of patients together with the severe chronic periodontitis patients represents the individuals in stages III &VI. The low recorded percentages of periodontitis in these two stages could be referred to as this exclusion. This consideration should be taken in future studies implementing the new 2018 periodontal classification.

**Comment 2**:

“In addition, the study design did not include diabetes mellitus and smoking among the studied risk factors. One of the strongest points of the study is that the national common food and drinks as beans and tea were included among the risk factors.”

**Answer:**

The authors agree with the reviewer’s comment and because of this in the discussion section the following statement was written to clarify this “Even though the current work investigated the occurrence of periodontal diseases in correlation to different risk factors, important risk factors still need to be investigated such as smoking and glycosylated haemoglobin level.”

Sincerely,

Marwa Abbass

# Peer review: Reviewer response for version 1

**DOI:** 10.5256/f1000research.22315.r55206

**Published:** 2019-11-04

**Authors:** Reema Fayez Tayyem

**Affiliations:** 1 Department of Nutrition and Food Technology, Faculty of Agriculture, University of Jordan, Amman, Jordan

The present study aimed to evaluate the prevalence and the severity of periodontal disease and its correlation with different risk factors.

 The paper is well-written and presented paper. However, many concerns and questions should be addressed to be able to index this paper. 

 All the comments and suggestions are presented in the pdf attached. 

Is the work clearly and accurately presented and does it cite the current literature?

Yes

If applicable, is the statistical analysis and its interpretation appropriate?

I cannot comment. A qualified statistician is required.

Are all the source data underlying the results available to ensure full reproducibility?

No source data required

Is the study design appropriate and is the work technically sound?

Partly

Are the conclusions drawn adequately supported by the results?

Yes

Are sufficient details of methods and analysis provided to allow replication by others?

Yes

Reviewer Expertise:

Clinical and community nutrition.

I confirm that I have read this submission and believe that I have an appropriate level of expertise to confirm that it is of an acceptable scientific standard, however I have significant reservations, as outlined above.

# Response

**Published:** 2020-03-11

**Authors:** Marwa Abbass

**Affiliations:** Faculty of Dentistry, Cairo University, Egypt

Dear Reviewer:

Thanks for your comprehensive revision for the current article

These are the responses for your valuable comments

**Comment 1:**

“It should be among adults living in Cairo”

**Answer:** 

When designing the study the following points were agreed by the authors:

-Great Cairo encompasses more than 15 million, a large number of them are internal immigrants from different regions of Egypt.

-low and middle SES Egyptians are the main beneficiaries of the free services at the Faculty of Dentistry, Cairo University which is a primary care center in Egypt. --To provide diversity, adults attending 3 private care centers also were recruited in the study to make sure of a heterogeneous population sample.

The authors agree with the reviewer that the sample is a small convenient one and that the population at the great Cairo may differ from their companions in rural Upper Egypt areas.  

Therefore, the title has been modified by adding “a convenient sample of” to the adult Egyptian population as well as a limitation paragraph that has been added to the discussion section to represent the reviewer and the author’s point of view. 

**Revised text:**

**Title**

The occurrence of periodontal diseases and its correlation with different risk factors among a convenient sample of adult Egyptian population: a cross-sectional study

**Discussion**

Moreover, among the limitations of the present study is the small and convenient sample recruited from adults attending the free dental clinic at the Faculty of Dentistry and three private clinics at great Cairo. Although, a large number of great Cairo residences are internal immigrants from different regions of Egypt, including other geographical regions from Egypt may provide a better and accurate representation of the Egyptian population.

**Comment 2:**

“Is the present study population-based survey? Did the authors include patients from all the geographical areas in Egypt?”

**Answer:**

Refer to the answer of comment #1

**Revised text:**

Refer to the revised text of comment #1

**Comment 3:**

I don't think the authors really measured the prevalence of **periodontitis**. They rather estimated the occurrence of **periodontitis** among the conveniently selected group.

**Answer:**

The authors agree with the reviewer and appreciate this point of view, therefore, the statement “a convenient sample of” has been added to the title as well as the term “prevalence” has been substituted by the term “occurrence” in the whole manuscript. 

**Revised Text:**

**Title**

The occurrence of periodontal diseases and its correlation with different risk factors among a convenient sample of adult Egyptian population: a cross-sectional study

**Comment 4:**

“and crackers”

**Answer:**

Rewarding has been performed according to the reviewer's recommendations.

**Revised Text:**

Periodontitis was positively correlated with age, carbohydrates other than bread, grains, and crackers, as well as caffeinated drinks

**Comment 5:**

“as well as”

**Answer:**

Rewarding has been performed according to the reviewer's recommendations.

**Revised Text:**

Periodontitis was positively correlated with age, carbohydrates other than bread, grains, and crackers, as well as caffeinated drinks

**Comment 6:**

“can be defined **or **is defined”

**Answer:**

Rewarding has been performed according to the reviewer's recommendations.

**Revised Text:**

Periodontitis is defined as a chronic, progressive inflammatory disease affecting the periodontium surrounding the tooth.

**Comment 7:**

“what do you mean by this?”

**Answer:**

Following the reviewer’s recommendation, clarification for this point has been performed.

**Revised Text:**

Therefore, the aim of the present study is to investigate the incidence of periodontal diseases in correlation with the risk factors amongst a convenient sample from the Egyptian population.

**Comment 8:**

“If all the patients from Cairo, then how you generalize the results for all the Egyptian population?”

**Answer:**

When designing the study the following points were agreed by the authors:

-Great Cairo encompasses more than 15 million, a large number of them are internal immigrants from different regions of Egypt.

-low and middle SES Egyptians are the main beneficiaries of the free services at the Faculty of Dentistry, Cairo University which is a primary care center in Egypt.

-To provide diversity, adults attending 3 private care centers also were recruited in the study to make sure of a heterogeneous population sample.

The authors agree with the reviewer that the sample is a small convenient one. In the manuscript the sentence “Convenience sample was utilized in this study” has been used to describe the included sample.

The authors modified the title by adding “a convenient sample of” to the adult Egyptian population as well as a limitation paragraph that has been added to the discussion section to represent the reviewer’s point of view. 

**Revised Text:**

**Title**

The occurrence of periodontal diseases and its correlation with different risk factors among a convenient sample of adult Egyptian population: a cross-sectional study

**Discussion**

Moreover, among the limitations of the present study is the small and convenient sample recruited from adults attending the free dental clinic at Faculty of Dentistry and three private clinics at great Cairo. Although, a large number of great Cairo residences are internal immigrants from different regions of Egypt, including other geographical regions from Egypt may provide a better and an accurate representation of the Egyptian population.

**Comment 9:**

“I think this number of patients is not enough to say it is a population study!!!”

**Answer:**

Refer to answer of comment #8

**Revised Text:**

Refer to the revised text of comment #8

**Comment 10:**

**“**number”

**Answer:**

Rewarding has been performed according to the reviewer's recommendations.

**Comment 11:**

**“**were”

**Answer:**

Rewarding has been performed according to the reviewer's recommendations.

**Revised Text:**

In this study, positive correlations were found between calculus, periodontitis, and age.

# Response

**Published:** 2020-04-06

**Authors:** Reema Tayyem

**Affiliations:** the university of Jordan, Jordan

All the requested corrections are addressed in a satisfactory manner.
